# Synthesis and Application of New Salan Titanium Complexes in the Catalytic Reduction of Aldehydes

**DOI:** 10.3390/molecules27206821

**Published:** 2022-10-12

**Authors:** Joana Hipólito, Ana M. Martins, Luis G. Alves

**Affiliations:** 1 Centro de Química Estrutural, Instituto Superior Técnico, Universidade de Lisboa, Av. Rovisco Pais 1, 1049-001 Lisboa, Portugal; 2 Centro de Química Estrutural, Associação do Instituto Superior Técnico para a Investigação e Desenvolvimento, Av. Rovisco Pais 1, 1049-003 Lisboa, Portugal

**Keywords:** salan ligands, titanium complexes, aldehyde reduction, homogeneous catalysis

## Abstract

Complexes of formula [(H_2_N_2_O_2_)TiCl_2_] and [(H_2_N_2_O_2_)Ti(O^i^Pr)_2_] (H_2_N_2_O_2_H_2_ = HOPh’CH_2_NH(CH_2_)_2_NHCH_2_Ph’OH, where Ph’ = 2,4-(CMe_2_Ph)C_6_H_2_) were synthesized by the reaction of the salan ligand precursor H_2_N_2_O_2_H_2_ with TiCl_4_ and Ti(O^i^Pr)_4_, respectively, in high yields. The dichlorido complex [(H_2_N_2_O_2_)TiCl_2_] revealed to be an efficient catalyst for the reduction of benzaldehyde in toluene. Full conversion was observed after 24 h at 55 °C in THF. The same catalyst also converted phenylacetaldehyde and hydrocinnamaldehyde into the corresponding alkanes quantitatively.

## 1. Introduction

Salan-based complexes are a well-established class of organometallic compounds that have been used for several catalytic applications [1,2,3,4]. Most of these studies have focused on olefin [5,6,7] and cyclic esters polymerization [8,9,10], olefin epoxidation [11,12,13], and sulfoxidation reactions [14,15,16,17]. Metal complexes supported by salan-type ligands have also been used in pinacol coupling reactions that are a convenient procedure for the reduction of carbonyl compounds, more specifically aromatic aldehydes [18,19]. The mechanism of these reactions is based on the coordination of the carbonyl oxygen atom of the substrates to the reduced metal centers, leading to the formation of radical pinacolate intermediates that upon dimerization form 1,2-diols through the formation of new C-C bonds. In the presence of low-valent metal species, deoxygenation can occur to yield the corresponding alkenes, but no formation of alkanes is observed [20,21]. The reduction of aldehydes and ketones to the corresponding alkanes is commonly achieved by the Wolff-Kishner reaction that proceeds through a hydrazone intermediate, resulting from the condensation of the carbonyl compound with hydrazine under very harsh basic conditions [22]. The Clemmensen reaction is another procedure widely used for the reduction of aldehydes and ketones to alkanes when substrates are sensitive to bases as this reaction takes place in strongly acidic media [23]. Carbonyl compounds may also be converted into the dithiane intermediate and reduced with Raney nickel to give the alkane product [24]. None of the above-described procedures are catalytic and present serious drawbacks of severe reaction conditions and toxic compounds. More recently, the catalytic deoxygenation of aldehydes and ketones to alkanes was accomplished by a limited number of well-defined metal complexes. The catalytic systems [MoCl_2_O_2_(H_2_O)_2_]/PhSiMe_3_ [25] and [Rh(μ-Cl)(CO)_2_]_2_/HSiMe_3_ [26] proved to be highly efficient for the deoxygenation of a large variety of ketones to alkanes. The cationic ruthenium hydride complex [(C_6_H_6_)(PCy_3_)(CO)RuH]BF_4_, in the presence of a phenol ligand, exhibited high catalytic activity for the reduction of carbonyl compounds with molecular hydrogen, yielding the corresponding alkanes [27]. In this work, we describe the catalytic reduction of aldehydes into the corresponding alkanes using salan-based Ti(IV) complexes under mild conditions.

## 2. Results and Discussion

### 2.1. Synthesis and Characterization

**Scheme 1:**
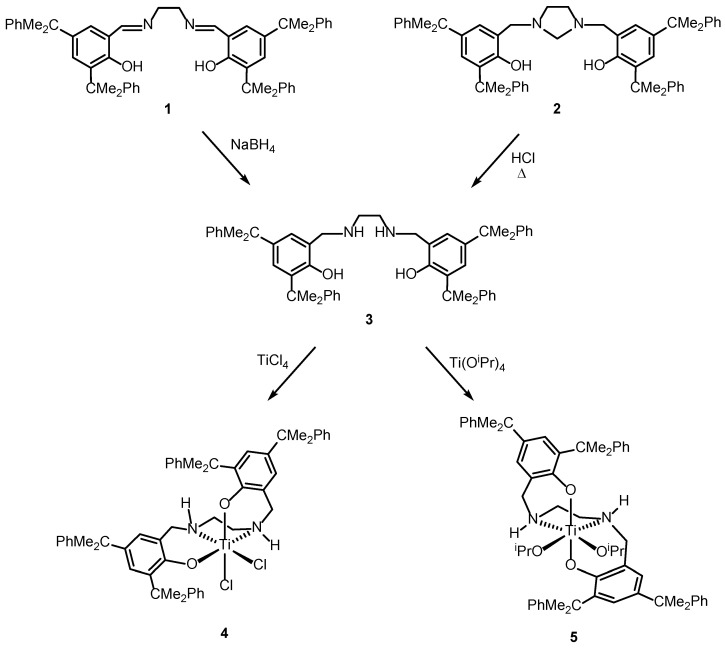
Synthetic rote for the preparation of compounds **1**–**5**.

The salan ligand precursor of the type H_2_N_2_O_2_H_2_, **3**, was prepared by acid hydrolysis of compound **2**, which is the main product of the Mannich coupling of 2,4-(CMe_2_Ph)_2_PhOH, formaldehyde and ethylene diamine [17]. Here, we describe an alternative protocol for the synthesis of **3** that involves the reduction of the salen species **1** with NaBH_4_, as depicted in Scheme 1. The yields of both reactions are similar, but having in consideration that the synthesis of **1** requires the preparation of 2-hydroxy-3,5-bis(2-phenylpropan-2-yl)benzaldehyde, which is not commercially available, the preparation of **3** by acid hydrolysis of **2** is a more convenient procedure. 

The reaction of **3** with TiCl_4_ and Ti(O^i^Pr)_4_ led to the formation, in high yields, of the new salan complexes [(H_2_N_2_O_2_)TiCl_2_], **4**, and [(H_2_N_2_O_2_)Ti(O^i^Pr)_2_], **5**, respectively, as shown in Scheme 1.

The ^1^H NMR spectrum of **4** (see Figure S5A) featuring two AX spin systems assigned to the NCH_2_C_PhO_ groups and two AX spin systems due the NCH_2_CH_2_N protons is consistent with an octahedral complex displaying a β-*cis* conformation as revealed by its solid-state molecular structure determined by single crystal X-ray diffraction (see discussion below) [28]. The four AX spin systems are directly related to four different carbon resonances corresponding to the NCH_2_C_PhO_ and NCH_2_CH_2_N fragments. The spectrum reveals two NH resonances at 5.06 and 0.62 ppm and a complex pattern in the aromatic region that are characteristic of the structure asymmetry. Cross-peaks between the NH protons and the CH_2_ diastereotopic protons of both NCH_2_CH_2_N and NCH_2_C_PhO_ moieties are observed in the ^1^H-^1^H COSY NMR spectrum (see Figure S5B). The ^1^H NMR spectrum of **5** (see Figure S6A) is much simpler than that of **4** showing the NCH_2_C_PhO_ protons as one AX spin system at 4.03 and 2.80 ppm and the methylene protons of the NCH_2_CH_2_N fragment as one multiplet that integrates to four protons due to their coupling with the NH protons that appear as a triplet at 0.36 ppm. These protons are shielded by the phenolate ring currents that are directed by the coordination of the oxygen to the titanium (2.48 ppm in **3** vs. 1.53–1.52 ppm in **5**). The ^1^H NMR spectrum also displays four CMe_2_Ph resonances and diastereotopic methyl resonances for the isopropoxido ligands. The ^13^C{^1^H} NMR spectra of **4** and **5** are in accordance with the pattern observed in the proton NMR spectra (see Figure S5C and Figure S6B, respectively).

**Figure 1:**
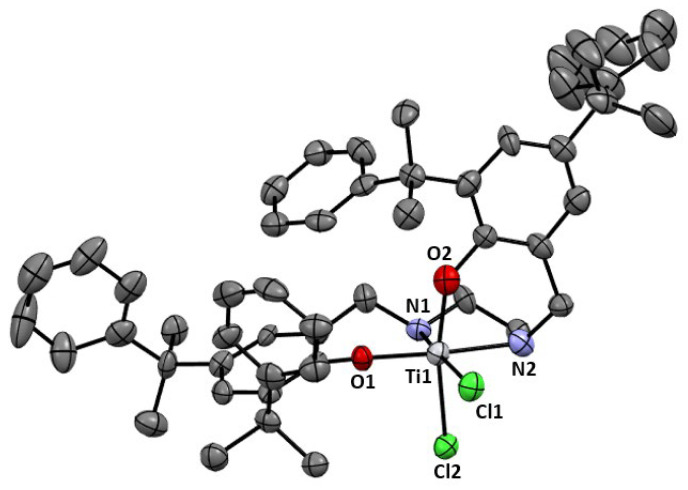
ORTEP diagram of [(H_2_N_2_O_2_)TiCl_2_], **4**, showing thermal ellipsoids at 35% probability level. Hydrogens atoms and co-crystallized solvent molecules were omitted for clarity. Selected bond lengths (Å) and angles (°): Ti(1)-N(1) 2.193(8), Ti(1)-N(2) 2.219(9), Ti(1)-O(1) 1.827(6), Ti(1)-O(2) 1.811(8), Ti(1)-Cl(1) 2.306(3), Ti(1)-Cl(2) 2.407(3); N(1)-Ti(1)-N(2) 75.6(3), N(1)-Ti(1)-O(1) 84.6(3), N(2)-Ti(1)-Cl(1) 96.2(2), O(1)-Ti(1)-Cl(1) 103.3(2), O(2)-Ti(1)-Cl(2) 166.3(2).

**Figure 2:**
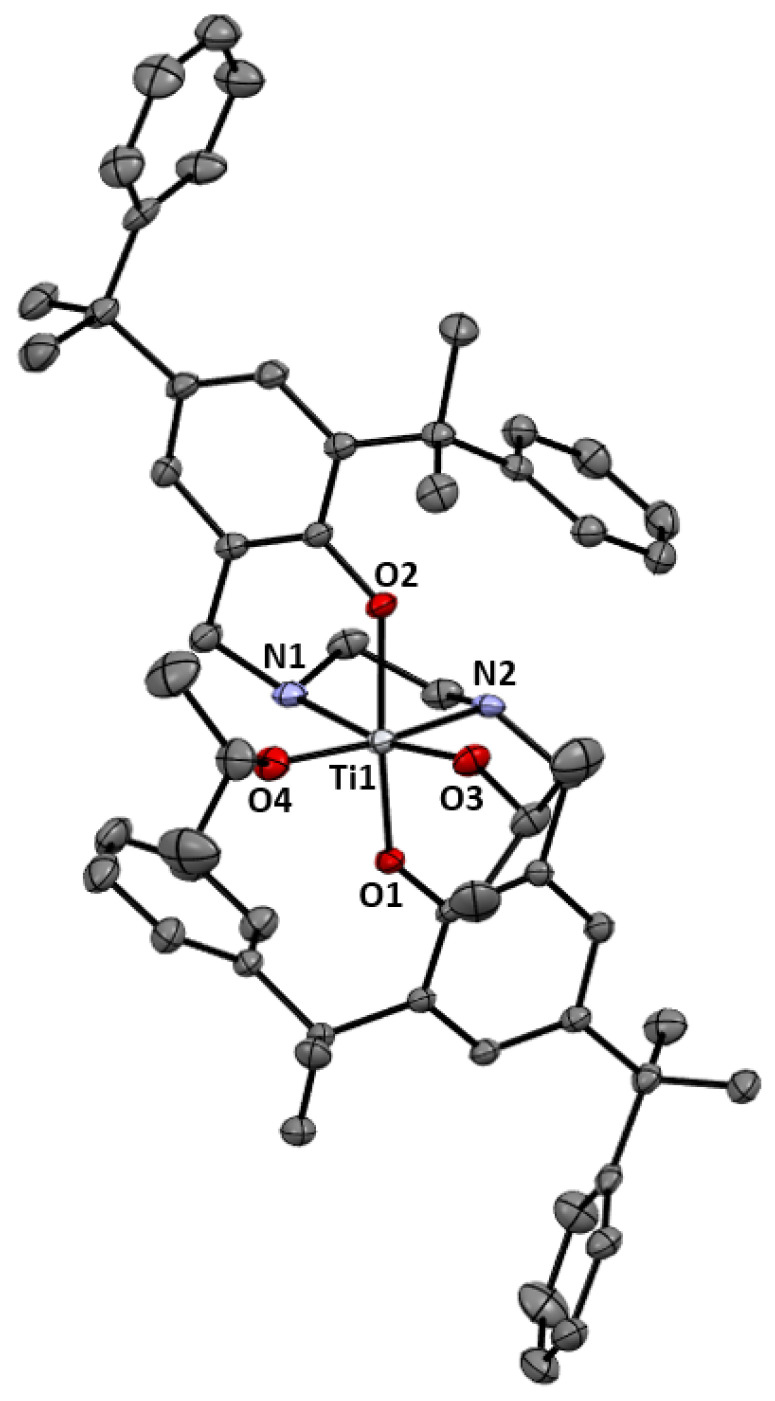
ORTEP diagram of [(H_2_N_2_O_2_)Ti(O^i^Pr)_2_], **5**, showing thermal ellipsoids at 40% probability level. Hydrogens atoms were omitted by clarity. Selected bond lengths (Å) and angles (°): Ti(1)-N(1) 2.267(2), Ti(1)-N(2) 2.277(2), Ti(1)-O(1) 1.902(2), Ti(1)-O(2) 1.911(2), Ti(1)-O(3) 1.809(2), Ti(1)-O(4) 1.788(2); N(1)-Ti(1)-N(2) 75.74(8), N(1)-Ti(1)-O(4) 93.25(8), N(2)-Ti(1)-O(3) 87.76(8), O(3)-Ti(1)-O(4) 103.35(8), O(1)-Ti(1)-O(2) 156.38(7).

Crystals of **4** and **5** suitable for single crystal X-ray diffraction were obtained from a concentrated diethyl ether solution at room temperature and from a toluene solution stored at −20 °C, respectively. Complex **4** crystallized in the tetragonal I4_1_/a space group and complex **5** crystallized in the triclinic P-1 space group. ORTEP diagrams of the solid-state molecular structures of **4** and **5** are shown in Figure 1 and Figure 2, respectively. 

Complexes **4** and **5** display distorted octahedral geometries around titanium centers. Complex **4** adopts a β-Δ-*cis* conformation with the equatorial plane defined by the Cl(1) atom and the N(1), N(2) and O(1) atoms of the salan ligand. The axial positions of the octahedron are occupied by the Cl(2) atom and by the O(2) atom of the salan ligand. On the other hand, complex **5** adopts a α-Δ-*cis* conformation with the equatorial plane defined by the N(1) and N(2) atoms of the salan ligand and the O(3) and O(4) atoms of the isopropoxido ligands. The axial positions are occupied by the O(1) and O(2) atoms of the salan ligand. The overall bond distances and angles determined for **4** and **5** are within the ranges reported for other titanium(IV) salan complexes described in the literature [29,30,31,32,33,34].

### 2.2. Catalytic Studies

Titanium complexes are efficient catalysts for the reductive coupling of carbonyl compounds leading to the corresponding diols. The catalysts of these reactions are formed in situ from the reduction of Ti(IV) precursors to Ti(III) species in the presence of suitable reducing agents [20,21]. Complexes **4** and **5** were evaluated for their catalytic potential in the reduction of aldehydes using manganese as the reducing agent and trimethylsilyl chloride (TMSCl), whose role is the regeneration of the catalytic cycle. Control reactions confirmed the lack of aldehyde reduction in the absence of the complexes, validating their role as catalysts (Table 1, entries 9 and 10). The radical nature of the reaction was confirmed by the results obtained in the presence of 2,2,6,6-tetramethyl-1-piperidinyloxy (TEMPO) (Table 1, entry 3), which blocks the reaction. The results, listed in Table 1, show that in the correct experimental conditions, the main products of these reactions are alkanes that result from the hydrogenation of the C=O group, even without the addition of hydrogen. This issue is tentatively discussed below.

Table 1 shows that complex **4** is the most active catalyst leading to almost quantitative conversion of benzaldehyde in toluene (Table 1, entries 4 and 5). The highest conversion of benzaldehyde in toluene was observed after 24 h at 55 °C in THF (Table 1, entry 5). At 30 °C, the conversion of benzaldehyde was also very high, but the selectivity towards toluene was lower, as benzyl alcohol and 1,2-diphenylethane-1,2-diol remained after 24 h (Table 1, entry 1). A comparison of the results of entries 1, 4, and 5 attests to the importance of temperature in the selectivity in toluene and shows that at 55 °C, the reaction was essentially completed after 7 h. The importance of THF as the solvent is likely related to the formation of hydrogen in the reaction medium (see below). The formation of benzyl alcohol as an intermediate product of the reduction of benzaldehyde in toluene was confirmed by an independent assay where it was used as the substate. Full conversion of benzyl alcohol in toluene was observed after 4 h at 55 °C. Aiming to evaluate the reduction ability of the system to non-aromatic aldehydes, phenylacetaldehyde and hydrocinnamaldehyde were tested under the optimal conditions. The reduction of both aldehydes into the corresponding alkanes was quantitatively achieved after 24 h at 55 °C using **4** as the catalyst (Table 2, entries 1 and 2).

**Figure 3:**
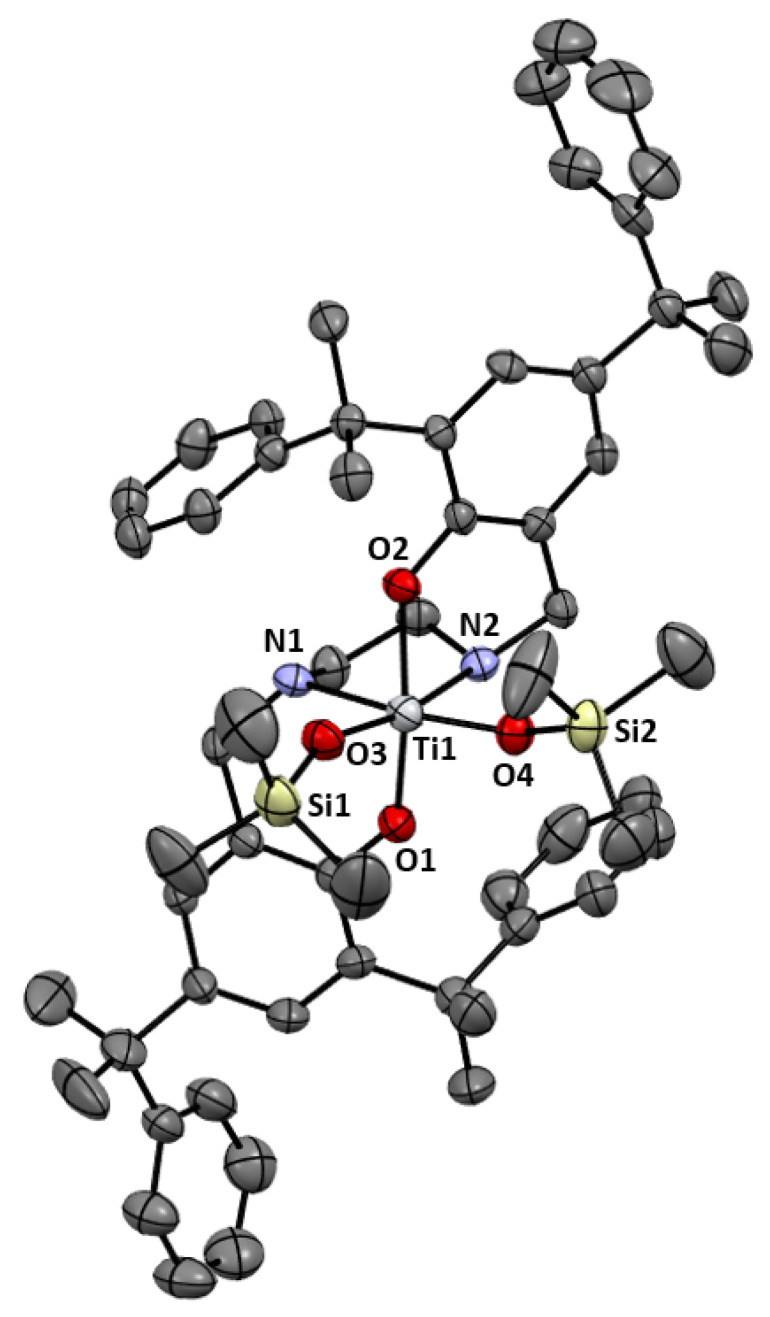
ORTEP diagram of [(H_2_N_2_O_2_)Ti(OSiMe_3_)_2_], **7** showing thermal ellipsoids at 35% probability level. Hydrogens atoms were omitted by clarity. Selected bond lengths (Å) and angles (°): Ti(1)-N(1) 2.252(4), Ti(1)-N(2) 2.257(4), Ti(1)-O(1) 1.882(3), Ti(1)-O(2) 1.899(3), Ti(1)-O(3) 1.820(3), Ti(1)-O(4) 1.826(3); N(1)-Ti(1)-N(2) 76.2(2), N(1)-Ti(1)-O(3) 91.2(2), N(2)-Ti(1)-O(4) 88.3(2), O(3)-Ti(1)-O(4) 104.3(2), O(1)-Ti(1)-O(2) 156.4(1).

Aiming to gain further insights into the catalytic system, the reaction of TiCl_3_(THF)_3_ and H_2_N_2_O_2_Na_2_, **6**, was carried out in THF. It was observed that the green solution that initially formed upon the mixture of reagents turned orange along time with the concomitant formation of hydrogen, revealing that [(H_2_N_2_O_2_)TiCl] is not stable in THF. In catalytic conditions (i.e., in the presence of Mn and TMSCl), the Ti(III) catalyst reacts with the substrate and also with THF to produce the corresponding alkane along with hydrogen. At the end of the catalytic reaction, a few yellow crystals of a new salan complex were obtained from the solution. This compound could be identified by single crystal X-ray diffraction as [(H_2_N_2_O_2_)Ti(OSiMe_3_)_2_], **7**, which displays two siloxane ligands, possibly formed from the reaction of an intermediate {Ti-O} species with TMSCl. The solid-state molecular structure of **7** (see Figure 3) shows an octahedral complex displaying an α-Λ-cis conformation with the equatorial plane defined by the N(1) and N(2) atoms of the salan ligand and the O(3) and O(4) atoms of the siloxane ligands. The axial positions are occupied by the O(1) and O(2) atoms of the salan ligand. The overall bond distances and angles determined for **7** are within the ranges reported for other titanium(IV) salan complexes described in the literature [29,30,31,32,33,34].

## 3. Materials and Methods

### 3.1. General Considerations

Commercial NaH (60% dispersion in mineral oil) was washed several times with n-hexane and dried under vacuum. TMSCl was freshly distilled under nitrogen before use. All other reagents were commercial grade and used without purification. All manipulations were performed under an atmosphere of dry oxygen-free nitrogen by means of standard Schlenk and glovebox techniques. Solvents were pre-dried using 4 Å molecular sieves and refluxed over sodium-benzophenone (diethyl ether, THF and toluene) or CaH_2_ (n-hexane) under an atmosphere of N_2_ and collected by distillation. Deuterated solvents were dried with 4 Å molecular sieves and freeze-pump-thaw degassed prior to use. NMR spectra were recorded in a Bruker AVANCE II 300 MHz or 400 MHz spectrometers, at 296 K, referenced internally to residual proton-solvent (^1^H) or solvent (^13^C) resonances, and reported relative to tetramethylsilane (0 ppm). 2D NMR experiments such as ^1^H-^13^C HSQC and ^1^H-^1^H COSY were performed in order to make all the assignments. Elemental analyses were carried out at the Laboratório de Análises do IST using an EA110CE automatic analyzer instrument. The analysis of the products obtained in catalytic reactions was conducted by HPLC using a Jasco system equipped with a Daicel Chiralpak IA column, an 870-UV Intelligent UV-Vis detector, two 880-PU Intelligent HPLC Pumps, a 2-line degasser 880-51 and a Rheodyne 725i injector (5 μL). The system uses Borwin software for data acquisition and analysis.

### 3.2. Synthesis and Characterization of the Compounds

#### 3.2.1. 2-Hydroxy-3,5-Bis(2-Phenylpropan-2-yl)Benzaldehyde

SnCl_4_ (0.6 mL, 5.0 mmol) was added dropwise, under nitrogen, to a mixture of 2,4-bis(α,α-dimethylbenzyl)phenol (16.52 g, 50.0 mmol) and tributylamine (4.8 mL, 20.0 mmol) in toluene (10 mL) for 15 min until white fumes disappeared. Paraformaldehyde (3.30 g, 110 mmol) was added in a single portion and the mixture was heated at 100 °C for 10 h. After cooling to room temperature, water (20 mL) was added, and the residue was extracted with diethyl ether (6 × 100 mL). The combined organic extracts were dried over MgSO_4_ anhydrous, filtered, and evaporated to dryness. White crystalline material was obtained from a concentrated hexane solution at −20 °C. Yield: 8.4% (1.52 g, 4.20 mmol). ^1^H NMR (CDCl_3_, 400.1 MHz, 296 K): δ (ppm) 11.26 (s, 1H, C(H)O), 9.75 (s, 1H, OH), 7.51 (s, 1H, CH_PhO_), 7.32–7.13 (overlapping, 11H total, CH_Ph_ and CH_PhO_), 1.72 (s, 6H, C(CH_3_)_2_), 1.64 (s, 6H, C(CH_3_)_2_). ^13^C{^1^H} NMR (CDCl_3_, 100.6 MHz, 296 K): δ (ppm) 197.0 (C(H)O), 158.8 (HOC_PhO_), 153.5 (C_PhO_), 150.1 (C_Ph_), 149.8 (C_Ph_), 141.4 (C_PhO_), 137.3 (C_PhO_), 133.9 (CH_PhO_), 129.6 (CH_PhO_), 128.3 (CH_Ph_), 128.0 (CH_Ph_), 126.8 (CH_Ph_), 126.1 (CH_Ph_), 125.7 (CH_Ph_), 125.5 (CH_Ph_), 42.7 (C(CH_3_)_2_), 42.1 (C(CH_3_)_2_), 30.9 (C(CH_3_)_2_), 29.3 (C(CH_3_)_2_). Anal. calcd. for C_25_H_26_O_2_.(H_2_O)_0.5_: C, 81.71; H, 7.41.Found: C, 82.36; H, 7.11.

#### 3.2.2. 6,6′-((1E,1′E)-(Ethane-1,2-Diylbis(Azaneylylidene))Bis(methaneylylidene))Bis(2,4-Bis(2-Phenylpropan-2-yl)Phenol), **1**

A concentrated solution of ethylenediamine (0.6 mL, 0.6 mmol) (1M in methanol) was diluted in 10 mL of methanol and slowly added to a solution of 2-hydroxy-3,5-bis(2-phenylpropan-2-yl)benzaldehyde (0.41 g, 1.10 mmol) in methanol (15 mL). The mixture was heated at 50 °C with vigorous stirring for 15 min. The yellow precipitate formed was filtered, washed with cold ethanol and dried under vacuum. Yield: 75% (0.32 g, 0.43 mmol). ^1^H NMR (CDCl_3,_ 300.1 MHz, 296 K): δ (ppm) 13.25 (s, 2H, OH), 8.22 (s, 2H, N=CH), 7.35 (d, 2H, ^4^*J*_H-H_ = 3 Hz, CH_PhO_), 7.33–7.14 (overlapping, 20H total, CH_Ph_), 7.03 (d, 2H, ^4^*J*_H-H_ = 3 Hz, CH_PhO_), 3.71 (s, 4H, NCH_2_CH_2_N), 1.74 (s, 12H, C(CH_3_)_2_), 1.69 (s, 12H, C(CH_3_)_2_). ^13^C{^1^H} NMR (CDCl_3_, 75.5 MHz, 296 K): δ (ppm) 167.1 (N=CH), 157.8 (HOC_PhO_), 150.9 (C_Ph_), 150.8 (C_Ph_), 139.7 (C_PhO_), 136.2 (CH_PhO_), 129.3 (CH_Ph_), 128.2 (CH_Ph_), 127.9 (CH_Ph_), 126.9 (CH_Ph_), 125.8 (CH_Ph_), 125.7 (CH_Ph_), 125.2 (CH_PhO_), 118.0 (NCHC_PhO_), 59.7 (NCH_2_CH_2_N), 42.6 (C(CH_3_)_2_), 42.3 (C(CH_3_)_2_), 31.1 (C(CH_3_)_2_), 29.5 (C(CH_3_)_2_). Anal. calcd. for C_52_H_56_N_2_O_2_.(C_2_H_5_OH): C, 82.40; H, 7.94; N, 3.56. Found: C, 82.45; H, 7.54; N, 3.81.

#### 3.2.3. 6,6′-(Imidazolidine-1,3-Diylbis(Methylene))Bis(2,4-Bis(2-Phenylpropan-2-yl)Phenol), **2**

Ethylenediamine (0.90 g, 15.00 mmol) and an aqueous solution of formaldehyde (3.2 mL, 43.50 mmol) (37% in water) were added to a solution of 2,4-bis(2-phenylpropane-2-yl)phenol (10.90 g, 33.00 mmol) in ethanol (100 mL). The reaction mixture was stirred under reflux for 48 h. The obtained white precipitate was filtered, washed with cold ethanol and dried in vacuum. Yield: 82% (7.74 g, 10.2 mmol). Crystals of **2** suitable for single crystal X-ray diffraction were obtained from a concentrated benzene solution at room temperature. ^1^H NMR (CDCl_3_, 300.1 MHz, 296 K): δ (ppm) 10.18 (br, 2H, OH), 7.33–7.23 (overlapping, 22H total, CH_Ph_ and CH_PhO_), 6.75 (s, 2H, CH_PhO_), 3.64 (s, 4H, NCH_2_C_PhO_), 3.26 (s, 2H, NCH_2_N), 2.70 (s, 4H, NCH_2_CH_2_N), 1.76 (s, 12H, C(CH_3_)_2_), 1.71 (s, 12H, C(CH_3_)_2_). ^13^C{^1^H} NMR (CDCl_3_, 75.5 MHz, 296 K): δ (ppm) 153.5 (HOC_PhO_), 151.5 (C_Ph_), 151.3 (C_Ph_), 140.2 (C_PhO_), 135.3 (C_PhO_), 128.0 (CH_Ph_), 127.8 (CH_Ph_), 126.8 (CH_Ph_), 125.6 (CH_Ph_), 125.5 (CH_Ph_), 125.3 (CH_Ph_), 125.2 (CH_PhO_), 125.1 (CH_PhO_), 121.2 (NCH_2_C_PhO_), 74.2 (NCH_2_N), 58.6 (NCH_2_C_PhO_), 51.1 (NCH_2_CH_2_N), 42.5 (C(CH_3_)_2_), 42.5 (C(CH_3_)_2_), 31.0 (C(CH_3_)_2_), 29.5 (C(CH_3_)_2_). Anal. calcd. for C_53_H_60_N_2_O_2_: C, 84.08; H, 7.99; N, 3.70. Found: C, 84.00; H, 8.02; N, 3.75.

#### 3.2.4. 6,6′-((Ethane-1,2-Diylbis(Azanediyl))Bis(Methylene))Bis(2,4-Bis(2-Phenylpropan-2-yl)phenol), H_2_N_2_O_2_H_2_, **3**

Compound **1** (0.16 g, 0.21 mmol) was dissolved in a 1:1 mixture of methanol and chloroform (20 mL). The solution was cooled in an ice bath and NaBH_4_ (0.16 g, 0.42 mmol) was slowly added with stirring. The mixture was allowed to react overnight at room temperature. The reaction mixture was cooled again in an ice bath and a saturated solution of ammonium chloride was gradually added until the bubbling stopped. The product was extracted with several portions of dichloromethane. The organic extracts were combined and dried over Na_2_SO_4_ anhydrous, filtered and evaporated to dryness. Yield: 61% (97 mg, 0.13 mmol). ^1^H NMR (CDCl_3_, 300.1 MHz, 296 K): δ (ppm) 7.30–7.11 (overlapping, 22H total, CH_Ph_ and CH_PhO_), 6.74 (s, 2H, CH_PhO_), 3.73 (s, 4H, NCH_2_C_PhO_), 2.48 (s, 4H, NCH_2_CH_2_N), 1.71 (s, 12H, C(CH_3_)_2_), 1.66 (s, 12H, C(CH_3_)_2_). ^13^C{^1^H} NMR (CDCl_3_, 75.5 MHz, 296 K): δ (ppm) 153.5 (HOC_PhO_), 151.6 (C_Ph_), 151.3(C_Ph_), 140.4 (C_PhO_), 135.5 (C_PhO_), 128.0 (CH_Ph_), 127.8 (CH_Ph_), 126.9 (CH_Ph_), 125.8 (CH_Ph_), 125.7 (CH_Ph_), 125.6 (CH_Ph_), 125.1 (CH_PhO_), 125.0 (CH_PhO_), 121.6 (NCH_2_C_PhO_), 52.3 (NCH_2_C_PhO_), 46.8 (NCH_2_CH_2_N), 42.6 (C(CH_3_)_2_), 42.1 (C(CH_3_)_2_), 31.1 (C(CH_3_)_2_), 29.5 (C(CH_3_)_2_). Anal. calcd. for C_52_H_60_N_2_O_2_: C, 83.83; H, 8.12; N, 3.76. Found: C, 83.25; H, 8.11; N, 3.60.

#### 3.2.5. [(H_2_N_2_O_2_)TiCl_2_], **4**

A toluene solution of **3** (0.30 g, 0.40 mmol) was slowly added to a 0.5 M solution of TiCl_4_ in toluene (0.9 mL, 0.4 mmol) at −20 °C. The temperature was allowed to rise slowly to 100 °C and the suspension was stirred under reflux overnight. The solvent was evaporated to dryness under vacuum. The orange product obtained was dissolved in diethyl ether. The solution was filtered and stored at room temperature leading to the formation of crystalline product that was collected by filtration. Yield: 89% (0.31 g, 0.35 mmol). Crystals of **4** suitable for single crystal X-ray diffraction were obtained from a diethyl ether solution at −20 °C. ^1^H NMR (C_6_D_6_, 400.1 MHz, 296 K): δ (ppm) 7.56 (d, 2H, ^3^*J*_H-H_ = 8 Hz, CH_Ph_), 7.39 (s, 1H, CH_PhO_), 7.36–7.34 (m, 2H, CH_PhO_ and CH_Ph_), 7.31–7.27 (overlapping, 5H total, CH_Ph_ and CH_PhO_), 7.25–7.18 (m, 4H, CH_Ph_), 7.13–6.99 (m, 4H, CH_Ph_), 6.89 (s, 1H, CH_PhO_), 6.82 (d, 2H, ^3^*J*_H-H_ = 8 Hz, CH_Ph_), 6.53 (s, 1H, CH_PhO_), 6.43–6.40 (m, 1H, CH_Ph_), 6.34 (t, 2H, ^3^*J*_H-H_ = 8 Hz, CH_Ph_), 5.16 (d, 1H, ^2^*J*_H-H_ = 12 Hz, NCH_2_C_PhO_), 5.05 (b, 1H, NH), 4.07 (t, 1H, ^2^*J*_H-H_ = 12 Hz, NCH_2_C_PhO_), 3.33 (d, 1H, ^2^*J*_H-H_ = 12 Hz, NCH_2_C_PhO_), 2.56–2.45 (overlapping, 2H total, NCH_2_CH_2_N and NCH_2_C_PhO_), 2.11 (s, 3H, (C(CH_3_)_2_), 2.06–2.01 (overlapping, 4H total, (C(CH_3_)_2_) and NCH_2_CH_2_N), 1.73 (s, 3H, C(CH_3_)_2_), 1.80 (t, 1H, ^2^*J*_H-H_ = 12 Hz, NCH_2_CH_2_N), 1.69 (d, 1H, ^2^*J*_H-H_ = 4 Hz, NCH_2_CH_2_N), 1.65 (m, 12H, C(CH_3_)_2_), 1.30 (s, 3H, C(CH_3_)_2_), 0.62 (b, 1H, NH). ^13^C{^1^H} NMR (C_6_D_6_, 100.6 MHz, 296 K) δ (ppm) 158.2 (OC_PhO_), 156.4 (OC_PhO_), 153.6 (C_Ph_), 151.2 (C_Ph_), 150.9 (C_Ph_), 150.4 (C_Ph_), 143.7 (C_PhO_), 143.6 (C_PhO_), 136.2 (C_PhO_), 135.8 (C_PhO_), 128.5 (CH_Ph_), 128.4 (CH_Ph_), 128.3 (CH_Ph_), 127.3 (NCH_2_C_PhO_), 127.2 (CH_PhO_), 127.1 (CH_Ph_), 127.0 (CH_Ph_), 126.8 (NCH_2_C_PhO_), 126.7 (CH_PhO_), 126.2 (CH_Ph_), 126.1 (CH_Ph_), 126.0 (CH_PhO_), 125.9 (CH_Ph_), 125.7 (CH_Ph_), 125.6 (CH_Ph_), 124.6 (CH_PhO_), 124.3 (CH_Ph_), 54.2 (NCH_2_C_PhO_), 54.1 (NCH_2_C_PhO_), 51.3 (NCH_2_CH_2_N), 46.6 (NCH_2_CH_2_N), 43.4 (C(CH_3_)_2_), 43.0 (C(CH_3_)_2_), 42.9 (C(CH_3_)_2_), 42.5 (C(CH_3_)_2_), 33.1 (C(CH_3_)_2_), 31.3 (C(CH_3_)_2_), 31.2 (C(CH_3_)_2_), 31.1 (C(CH_3_)_2_), 30.9 (C(CH_3_)_2_), 30.6 (C(CH_3_)_2_), 29.9 (C(CH_3_)_2_), 26.8 (C(CH_3_)_2_). Anal. calcd. for C_52_H_58_Cl_2_N_2_O_2_Ti: C, 72.47; H, 6.78; N, 3.25. Found: C, 72.80; H, 6.74; N, 3.00.

#### 3.2.6. [(H_2_N_2_O_2_)Ti(O^i^Pr)_2_], **5**

A THF solution of **3** (0.83 g, 1.00 mmol) was slowly added to a 1M solution of Ti(O^i^Pr)_4_ in toluene (1.1 mL, 1.1 mmol) in the same solvent. The solution was stirred for 16 h at room temperature. The yellow solution obtained was evaporated to dryness, and the residue was extracted with toluene. Evaporation of the solvent to dryness led to a yellow crystalline solid. Yield: 79% (0.72 g, 0.79 mmol). Crystals of **5** suitable for single crystal X-ray diffraction were grown from a toluene solution at −20 °C. ^1^H NMR (C_6_D_6_, 300.1 MHz, 296 K): δ (ppm) 7.55 (d, 2H, ^4^*J*_H-H_ = 2 Hz, CH_PhO_), 7.35–7.19 (overlapping, 12H total, CH_Ph_), 7.07–6.96 (overlapping, 6H total, CH_PhO_ and CH_Ph_), 6.85–6.80 (overlapping, 4H total, CH_Ph_), 4.67 (sept, 2H,*^3^J*_H-H_ = 6 Hz, OCH(CH_3_)_2_), 4.04 (dd, 2H, *^2^J*_H-H_ = 14 Hz, NCH_2_C_PhO_), 2.80 (dd, 2H, *^2^J*_H-H_ = 14 Hz, NCH_2_C_PhO_), 2.14 (s, 6H, C(CH_3_)_2_), 1.74 (d, 12H, ^4^*J*_H-H_ = 3 Hz, C(CH_3_)_2_), 1.57 (s, 6H, C(CH_3_)_2_), 1.53-1.52 (overlapping, 4H total, NCH_2_CH_2_N), 1.22 (d, 6H,*^3^J*_H-H_ = 6 Hz, (OCH(CH_3_)_2_), 1.19 (d, 6H,*^3^J*_H-H_ = 6 Hz, OCH(CH_3_)_2_), 0.36 (br, 2H, NH). ^13^C{^1^H} NMR (C_6_D_6_, 75.5 MHz, 296 K) δ (ppm) 158.3 (OC_PhO_), 153.9 (C_Ph_), 152.2 (C_Ph_), 138.1 (C_PhO_), 136.5 (C_PhO_), 128.4 (CH_Ph_), 127.2 (CH_Ph_), 126.8 (CH_Ph_), 126.6 (CH_PhO_), 125.9 (CH_Ph_), 124.2 (CH_PhO_), 124.1 (CH_Ph_), 123.5 (NCH_2_C_PhO_), 76.5 (OCH(CH_3_)_2_), 53.9 (NCH_2_C_PhO_), 42.8 (C(CH_3_)_2_), 42.1 (C(CH_3_)_2_), 46.2 (NCH_2_CH_2_N), 38.2 (C(CH_3_)_2_), 31.7 (C(CH_3_)_2_), 31.6 (C(CH_3_)_2_), 26.9 (OCH(CH_3_)_2_), 26.6 (OCH(CH_3_)_2_), 25.4 (C(CH_3_)_2_). Anal. calcd. for C_58_H_72_N_2_O_4_Ti: C, 76.63; H, 7.98; N, 3.08; Found: C, 76.10; H, 8.40; N, 3.16.

#### 3.2.7. H_2_N_2_O_2_Na_2_, **6**

A THF solution of compound **3** (0.77 g, 1.03 mmol) was added dropwise to suspension of NaH (56 mg, 2.48 mmol) in the same solvent. The mixture was heated at 50 °C with vigorous stirring for 4 h. The solvent was evaporated to dryness and the product isolated as a white solid. Yield: 64% (0.49 g, 0.66 mmol). ^1^H NMR (C_6_D_6_, 300.1 MHz, 296 K): δ (ppm) 7.57–7.54 (overlapping, 6H total, CH_Ph_ and CH_PhO_), 7.41 (d, 4H, ^3^*J*_H-H_ = 6 Hz, CH_Ph_), 7.23 (dd, 4H, CH_Ph_), 7.10–7.05 (overlapping, 8H total, CH_Ph_ and CH_PhO_), 7.23 (t, 2H, ^3^*J*_H-H_ = 6 Hz, CH_Ph_), 3.33–3.26 (overlapping, 12H total, C_4_H_8_O and NCH_2_C_PhO_), 2.05 (s, 4H, NCH_2_CH_2_N), 1.88 (s, 12H, C(CH_3_)_2_), 1.83 (s, 12H, C(CH_3_)_2_), 1.33 (s, 8H, C_4_H_8_O), −0.08 (b, 2H, NH). ^13^C{^1^H} NMR (C_6_D_6_, 75.5 MHz, 296 K): δ (ppm) 165.6 (NaOC_PhO_), 155.2 (C_Ph_), 153.5 (C_Ph_), 135.4 (C_PhO_), 130.5 (C_PhO_), 128.3 (CH_Ph_), 128.1 (CH_Ph_), 127.4 (CH_PhO_), 127.3 (NCH_2_C_PhO_), 125.5 (CH_Ph_), 125.4 (CH_Ph_), 125.1 (CH_Ph_), 124.4 (CH_PhO_), 67.8 (C_4_H_8_O), 52.6 (NCH_2_C_PhO_), 48.2 (NCH_2_CH_2_N), 43.1 (C(CH_3_)_2_), 42.6 (C(CH_3_)_2_), 32.0 (C(CH_3_)_2_), 25.7 (C_4_H_8_O). Anal. calcd. for C_52_H_58_N_2_Na_2_O_2_.(C_4_H_8_O)_4.5_: C, 75.30; H, 8.40; N, 2.65. Found: C, 74.98; H, 7.93; N, 3.01.

### 3.3. General Procedure for the Catalytic Reduction of Aldehydes

The preparation of the catalytic reaction mixtures was performed inside a dry oxygen-free nitrogen filled glovebox. In a typical run, a solution of the selected catalyst (0.025 mmol) was added to a suspension of Mn (3 mmol). The reaction mixture was stirred at room temperature for 30 min until the color changed from orange to green. TMSCl (1.5 mmol) was added to the mixture followed by the substrate addition. Solvent was added to make up a total volume of 4 mL. The vial containing the catalytic mixture was sealed and magnetically stirred, outside the glovebox, during the catalytic run at a given temperature (30 °C or 55 °C). Acetone (4 mL) and diphenylsulfone (1 mmol) were added in the end of the catalytic reaction. The analysis of the products was performed by HPLC using a UV detector operating at 220 nm. A solvent mixture of n-heptane/isopropanol (95:5) was used as the eluent with a flow rate of 1.0 mL.min^−1^.

### 3.4. General Procedure for Single Crystal X-ray Crystallography 

Suitable crystals of compounds **2**, **4**, **5**, and **7** were coated and selected in Fomblin^®^ oil under an inert atmosphere of nitrogen. Crystals were then mounted on a loop external to the glovebox environment and data collected using graphite monochromated Mo-Kα radiation (λ = 0.71073 Å) on a Bruker AXS-KAPPA APEX II diffractometer (Bruker AXS Inc., Madison, WI, USA) equipped with an Oxford Cryosystem open-flow nitrogen cryostat. Cell parameters were retrieved using Bruker SMART software and refined using Bruker SAINT on all observed reflections [35]. Absorption corrections were applied using SADABS [36]. The structures were solved by direct methods using SIR97 [37] and SIR2004 [38]. Structure refinement was conducted using SHELXL [39]. These programs are part of the WinGX software package version 1.80.01 [40] system of programs. Hydrogen atoms of the NH and OH groups were located in the electron density map. The other hydrogen atoms were inserted in calculated positions and allowed to refine in the parent carbon atoms. Compound **4** crystallized with disordered molecules of solvent in the asymmetric unit and thus the Squeeze/PLATON sequence [41] was applied as all attempts to model the disorder did not lead to acceptable solutions. The poor diffracting power and crystal quality of **2**, **4**, and **7** (as attested by the *R*_int_ values obtained) precluded the final refinement to lower the corresponding *R* values. The crystallographic and experimental details of data collection and crystal structure determinations are available in Table 3. 

## 4. Conclusions

New salan Ti(IV) complexes were synthesized, characterized and tested as catalysts for the reduction of aldehydes. The results obtained showed that in correct experimental conditions the main products are alkanes that result from the hydrogenation of the C=O group, even without the addition of hydrogen. Complex [(H_2_N_2_O_2_)TiCl_2_] was the most active catalyst converting benzaldehyde, phenylacetaldehyde and hydrocinnamaldehyde almost quantitatively after 24 h at 55 °C in THF.

## Figures and Tables

**Table 1:**
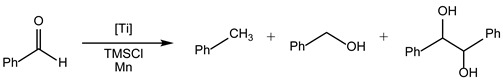
Catalytic data for the reduction of benzaldehyde ^a,b^.

Entry	Catalyst	Solvent	T (°C)	t (h)	Conv. (%)	Alkane (%)	Alcohol (%)	Diol (%)
1	**4**	THF	30	24	93	75	9	4
2	**4**	THF	55	4	82	78	0	2
3 ^c^	**4**	THF	55	4	55	3	0	0
4	**4**	THF	55	7	91	89	0	1
5	**4**	THF	55	24	94	92	0	1
6	**4**	EtOH	55	4	47	46	1	0
7	**4**	n-Bu_2_O/H_2_O	55	24	44	40	4	0
8	**5**	THF	30	24	75	69	5	0
9	**-**	THF	55	4	29	2	4	0
10	**-**	EtOH	55	4	3	3	0	0

^a^ 1 mmol benzaldehyde, 0.025 mmol catalyst, 1.5 mmol TMSCl, 3 mmol Mn, V_t_ = 4 mL; ^b^ Conversion and yield determined by HPLC; ^c^ Carried out in the presence of TEMPO.

**Table 2:**
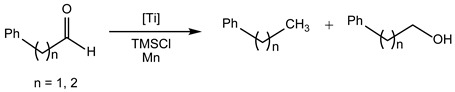
Substrate scope using **4** as the catalyst ^a,b^.

Entry	Substrate	t (h)	Conv. (%)	Alkane (%)	Alcohol (%)
1	PhCH_2_C(O)H	24	99	87	0
2	PhCH_2_CH_2_C(O)H	24	99	84	0

^a^ 1 mmol substate, 0.025 mmol catalyst, 1.5 mmol TMSCl, 3 mmol Mn, T = 55 °C, V_t_ = 4 mL THF; ^b^ Conversion determined by HPLC.

**Table 3:** Crystal data and structure refinement for complexes **4**, **5**, and **7**.

Compound	4_2_.(C_4_H_10_O)_3_	5	7
Empirical formula	C116 H146 Cl4 N4 O7 Ti2	C58 H72 N2 O4 Ti	C58 H76 N2 O4 Si2 Ti
Formula weight	1945.91	909.05	969.26
Temperature (K)	150 (2)	150 (2)	294 (2)
Crystal system, space group	Tetragonal, I4_1_/a	Triclinic, P-1	Triclinic, P-1
*a* (Å)	25.4100 (1)	11.664 (3)	10.315 (2)
*b* (Å)	25.4100 (1)	15.050 (6)	14.434 (4)
*c* (Å)	33.3570 (2)	16.062 (5)	19.035 (5)
α(º)	90	73.87 (2)	79.30 (1)
β(º)	90	86.84 (1)	86.65 (1)
γ(º)	90	67.29 (1)	88.76 (2)
Volume (Å^3^)	21537.6 (2)	2494.3 (15)	2779.9 (12)
Z	8	2	2
Calculated density (g m^−3^)	1.200	1.210	1.158
Absorption coefficient (mm^−1^)	0.303	0.220	0.242
*F* (000)	8304	976	1040
Crystal size (mm)	0.20 × 0.26 × 0.34	0.10 × 0.16 × 0.22	0.08 × 0.08 × 0.14
θ range for data collection (°)	1.007–25.576	3.163–27.046	1.978–26.558
Limiting indices	−30 ≤ *h* ≤ 30, −30 ≤ *k* ≤ 30, −37 ≤ *l* ≤ 40	−14 ≤ *h* ≤ 11, −18 ≤ *k* ≤ 14, −20 ≤ *l* ≤ 20	−12 ≤ *h* ≤ 12, −18 ≤ *k* ≤ 18, −23 ≤ *l* ≤ 23
Reflections collected/unique	117534/10101 [*R*_int_ = 0.2329]	26502/10473 [*R*_int_ = 0.0730]	62151/10227 [*R*_int_ = 0.3149]
Completeness to θ = 25.242	99.9	99.5	88.8
Data/restraints/parameters	10029/0/612	10473/0/606	10227/0/626
Goodness-of-fit on *F*^2^	1.519	0.961	0.842
Final *R* indices [*I* > 2*σ*(*I*)] ^a^	*R*_1_ = 0.1579, *wR*_2_ = 0.4442	*R*_1_ = 0.0558, *wR_2_* = 0.1186	*R*_1_ = 0.0897, *wR*_2_ = 0.2013
*R* indices (all data) ^a^	*R*_1_ = 0.2840, *wR*_2_ = 0.4777	*R*_1_ = 0.1105, *wR_2_* = 0.1365	*R*_1_ = 0.2545, *wR*_2_ = 0.2425
Largest diff. peak/hole (e Å^−3^)	1.176 and −0.592	0.379 and −0.720	0.748 and −0.469

^a^*R*_1_ = Σ ||F_0_| − |F_c_||/Σ |F_0_|; *wR*_2_ = {Σ[*w*(F^2^_0_ − F^2^_c_)^2^]/Σ[*w*(F^2^_0_)^2^]}^1/2^.

## Supplementary Materials

The following supporting information can be downloaded at: https://www.mdpi.com/article/10.3390/molecules27206821/s1. Figure S1 and Table S1: The single crystal X-ray diffraction analysis of compound **3**, Figures S2–S7: NMR spectra of compounds **1**–**6**, Figure S8: HPLC spectra of the benzaldehyde reduction products obtained in selected catalytic conditions. Data for structures **2**, **4**, **5** and **7** were deposited in the Cambridge Crystallographic Data Center (CCDC) under the deposit numbers 2205633-2205636 and can be obtained free of charge via http://www.ccdc.cam.ac.uk/conts/retrieving.html (accessed on 13 September 2022).

## Sample Availability

Samples of the compounds are available from the authors.
