# Supplementary material for: Synthesis and Application of New Salan Titanium Complexes in the Catalytic Reduction of Aldehydes
Source: Molecules. 2022 Oct 12;27(20):6821. doi: 10.3390/molecules27206821 (PMC9610537; doi:10.3390/molecules27206821)
Supplement: Supplementary file 1 [file molecules-27-06821-s001.zip › molecules-1943628-supplementary.pdf]

Synthesis and Application of New Salan Titanium Complexes in the Catalytic Reduction of Aldehydes

Joana Hipólito [1], Ana M. Martins [1,*] and Luis G. Alves [2,*]

[1] Centro de Química Estrutural, Instituto Superior Técnico, Universidade de Lisboa, Av. Rovisco Pais 1, 1049-001 Lisboa, Portugal

[2] Centro de Química Estrutural—Institute of Molecular Sciences, Associação do Instituto Superior Técnico para a Investigação e Desenvolvimento, Av. Rovisco Pais 1, 1049-003 Lisboa, Portugal

* Correspondence: ana.martins@tecnico.ulisboa.pt (A.M.M.); luis.g.alves@tecnico.ulisboa.pt (L.G.A.)

Single crystal X-ray diffraction analysis of compound 2

Crystals of compound **2** suitable for X-ray diffraction were obtained from a concentrated benzene solution at room temperature. The solid-state molecular structure of **2** is stabilized by the formation of intramolecular hydrogen bonds between the OH groups of the phenols and the N atoms of the amine moieties (see Figure SI1). Such interactions show O(1)-H(1O)···N(1) and O(2)-H(2O)···N(2) bond lengths of 2.06 and 1.92 Å, respectively, and define two 6-member heterocycles.

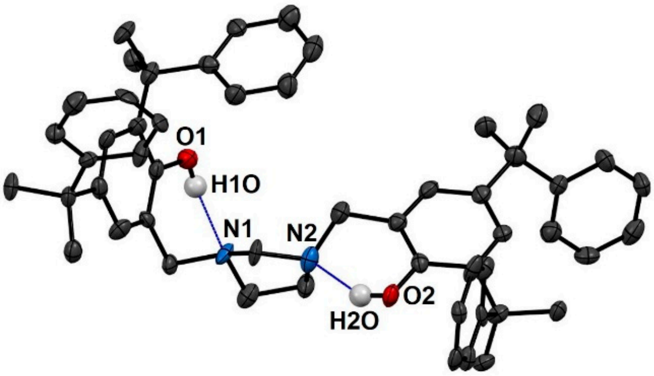

Figure S1. ORTEP diagram of **2** showing thermal ellipsoids at 40% probability level. Selected hydrogens atoms and co-crystallized solvent molecules were omitted for clarity.

Table S1 - Crystal data and structure refinement for compound **2**.

Compound	**2.(C$_6$H$_6$)$_{1.5}$**
Empirical formula	C62 H69 N2 O2
Formula weight	874.19
Temperature (K)	150(2)
Wavelength (Å)	0.71073
Crystal system, space group	Triclinic, P-1
a (Å)	10.802(7)
b (Å)	13.802(7)
c (Å)	17.804(9)
α(º)	72.03(3)
β(º)	89.71(4)
γ(º)	81.14(3)
Volume (Å^3)	2492(2)

Z	2
Calculated density (g m^{-3})	1.165
Absorption coefficient (mm^{-1})	0.069
F (000)	942
Crystal size (mm)	0.10 x 0.20 x 0.20
θ range for data collection (º)	1.571 – 25.835
Limiting indices	$-12 \leq h \leq 12$, $-15 \leq k \leq 16$, $-21 \leq l \leq 21$
Reflections collected/unique	19307/7916 [R_{int} = 0.2103]
Completeness to θ = 25.242	86.0
Data/restraints/parameters	7916/0/605
Goodness-of-fit on F^2	0.863
Final *R* indices [$I>2\sigma(I)$][a]	R_1 = 0.1055, wR_2 = 0.2183
R indices (all data)[a]	R_1 = 0.3046, wR_2 = 0.2781
Largest diff. peak/hole (e Å^{-3})	0.307 and -0.379

[a] $R_1 = \Sigma ||F_0| - |F_c|| / \Sigma |F_0|$; $wR_2 = \{\Sigma[w(F^2_0 - F^2_c)^2] / \Sigma[w(F^2_0)^2]\}^{1/2}$

NMR spectra of compounds 1-6

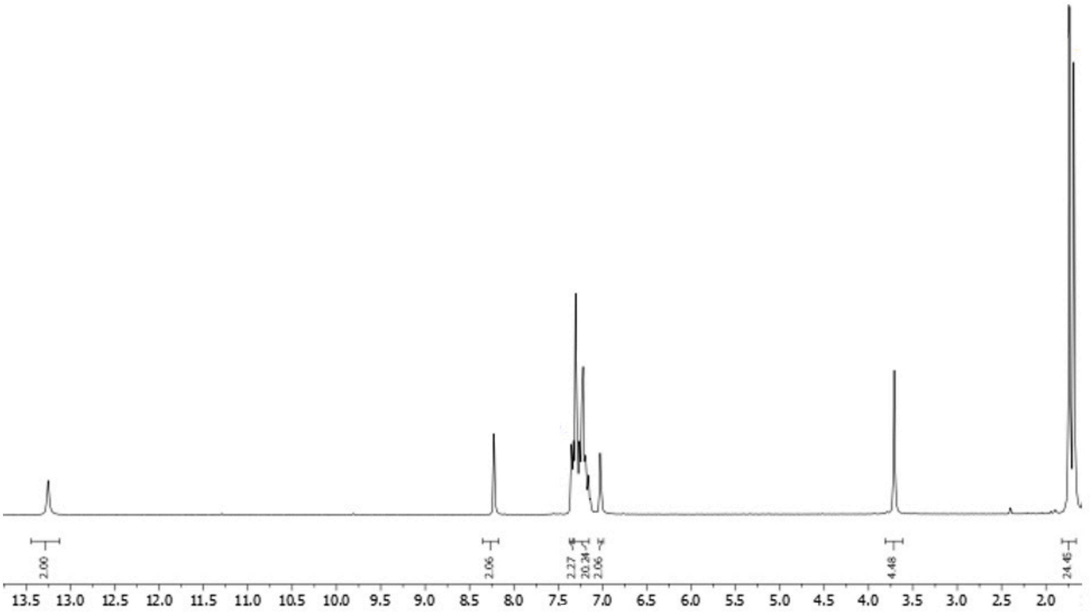

Figure S2A. ^1H NMR spectrum of **1** in $CDCl_3$.

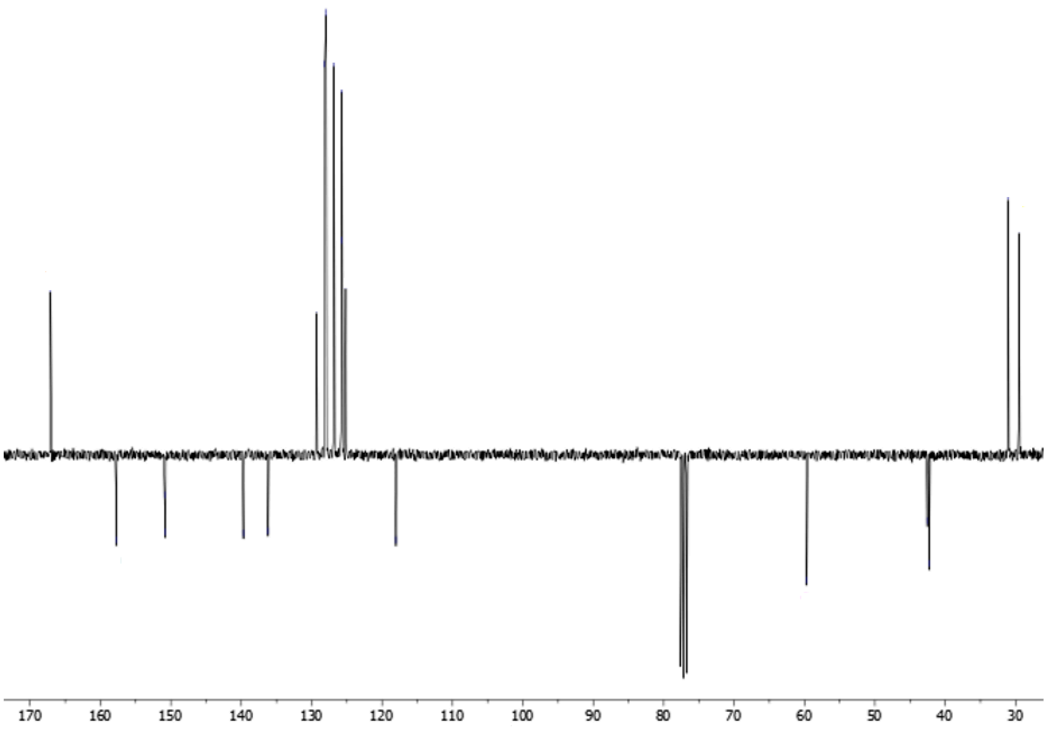

Figure S2B. $^{13}C\{^{1}H\}$ NMR spectrum of **1** in $CDCl_3$.

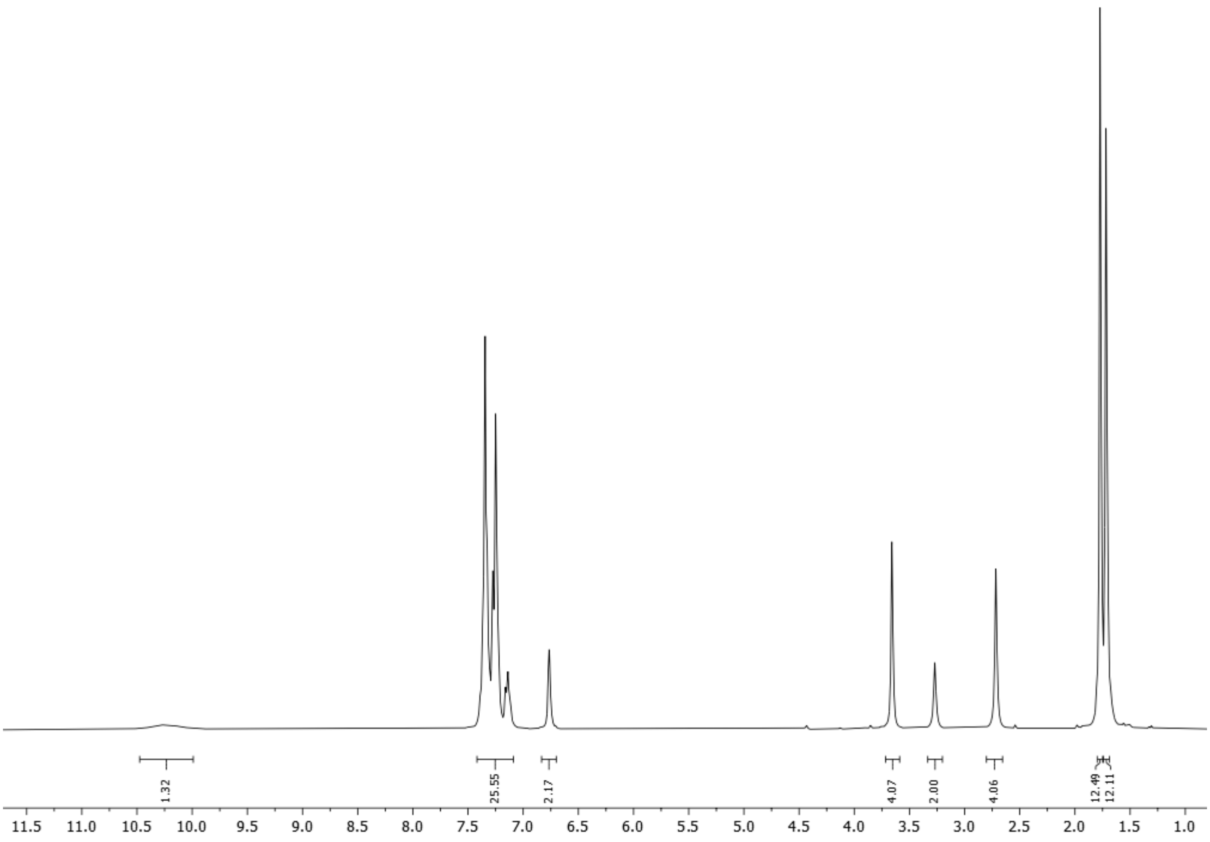

Figure S3A. ^{1}H NMR spectrum of **2** in $CDCl_3$.

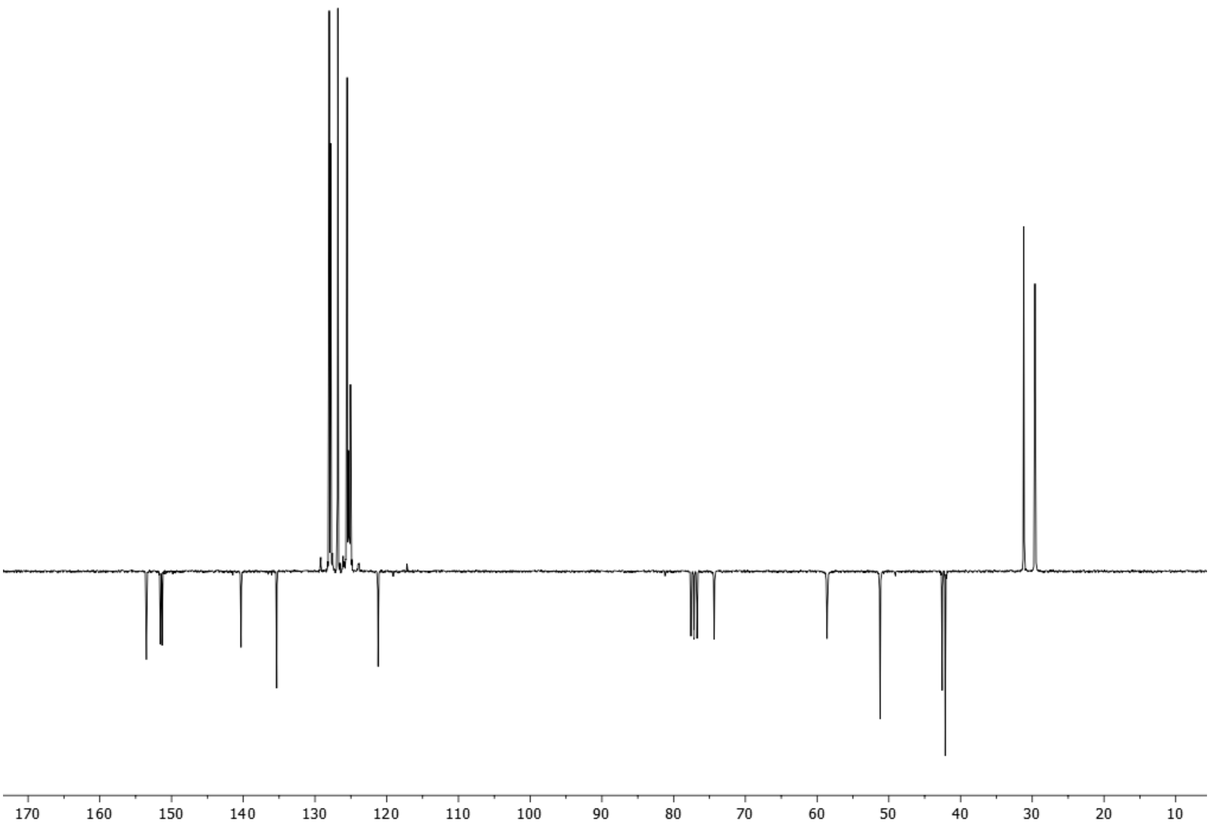

Figure S3B. $^{13}C\{^{1}H\}$ NMR spectrum of **2** in $CDCl_3$.

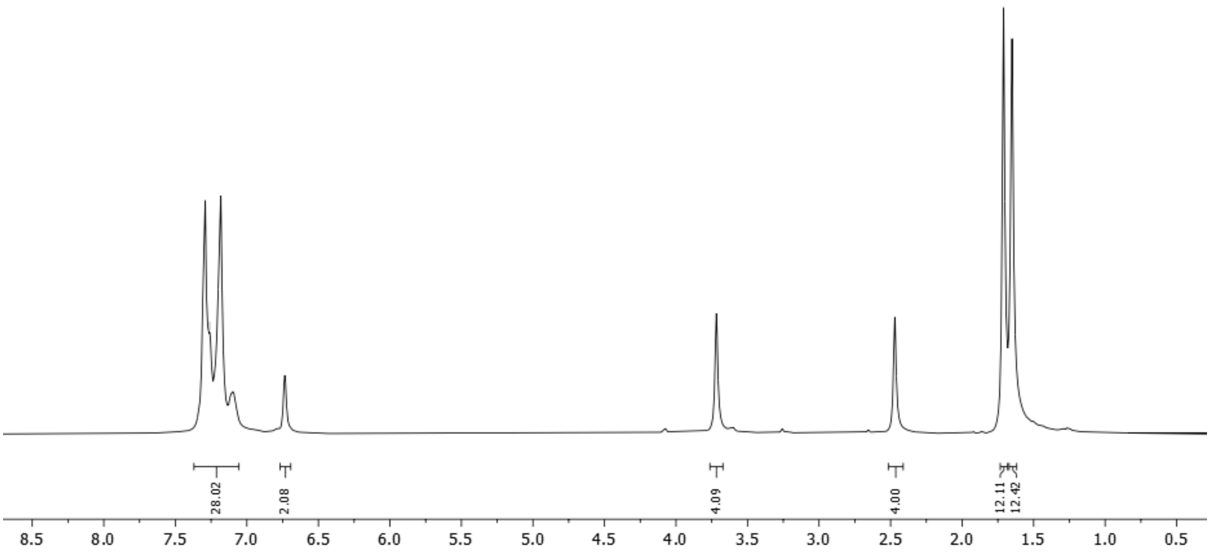

Figure S4A. ^{1}H NMR spectrum of **3** in $CDCl_3$.

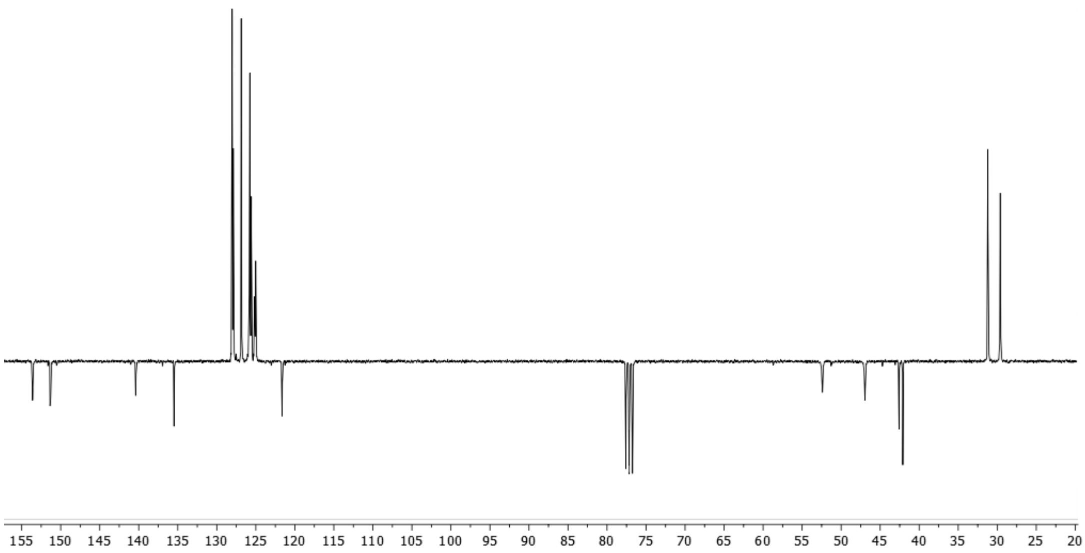

Figure S4B. $^{13}C\{^{1}H\}$ NMR spectrum of **3** in $CDCl_3$.

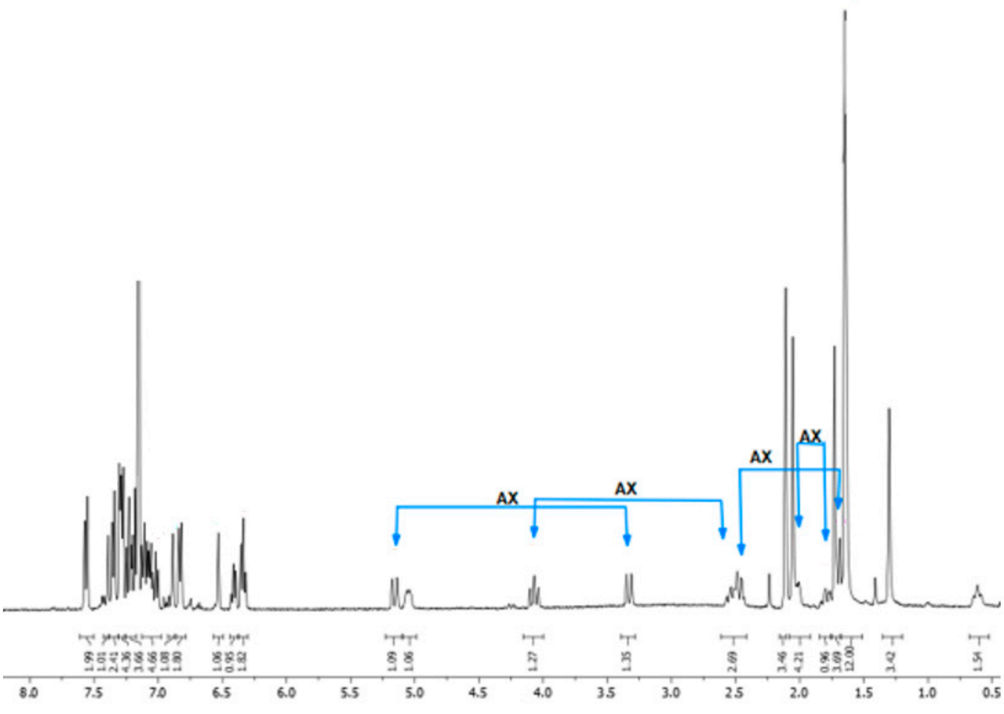

Figure S5A. ^{1}H NMR spectrum of **4** in C_6D_6.

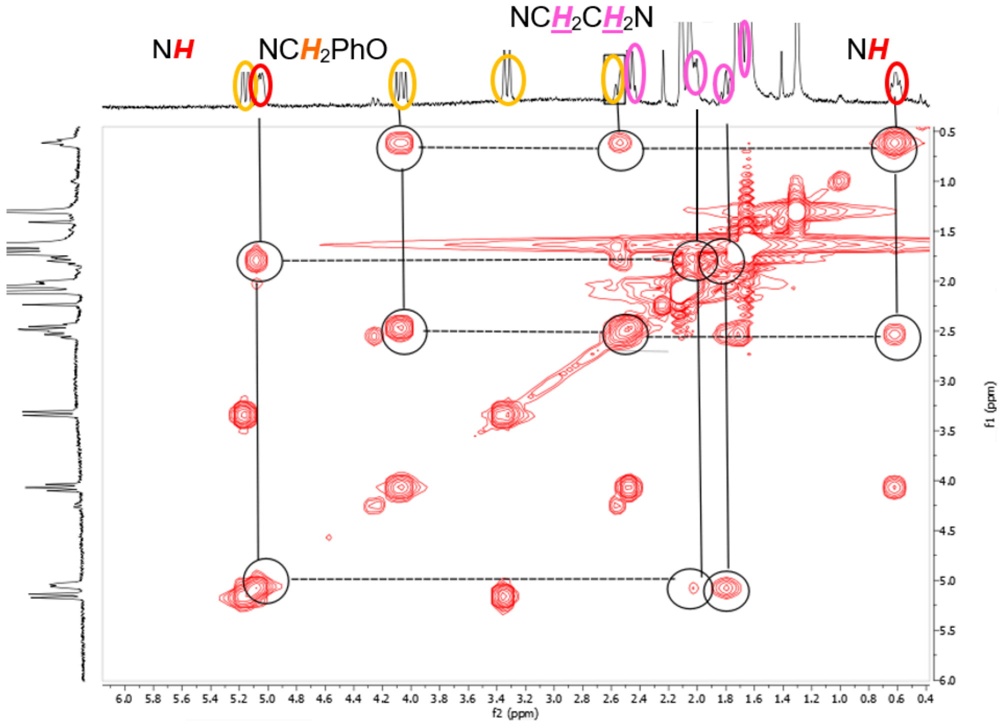

Figure S5B. ^{1}H-^{1}H COSY NMR spectrum of **4** in C_6D_6

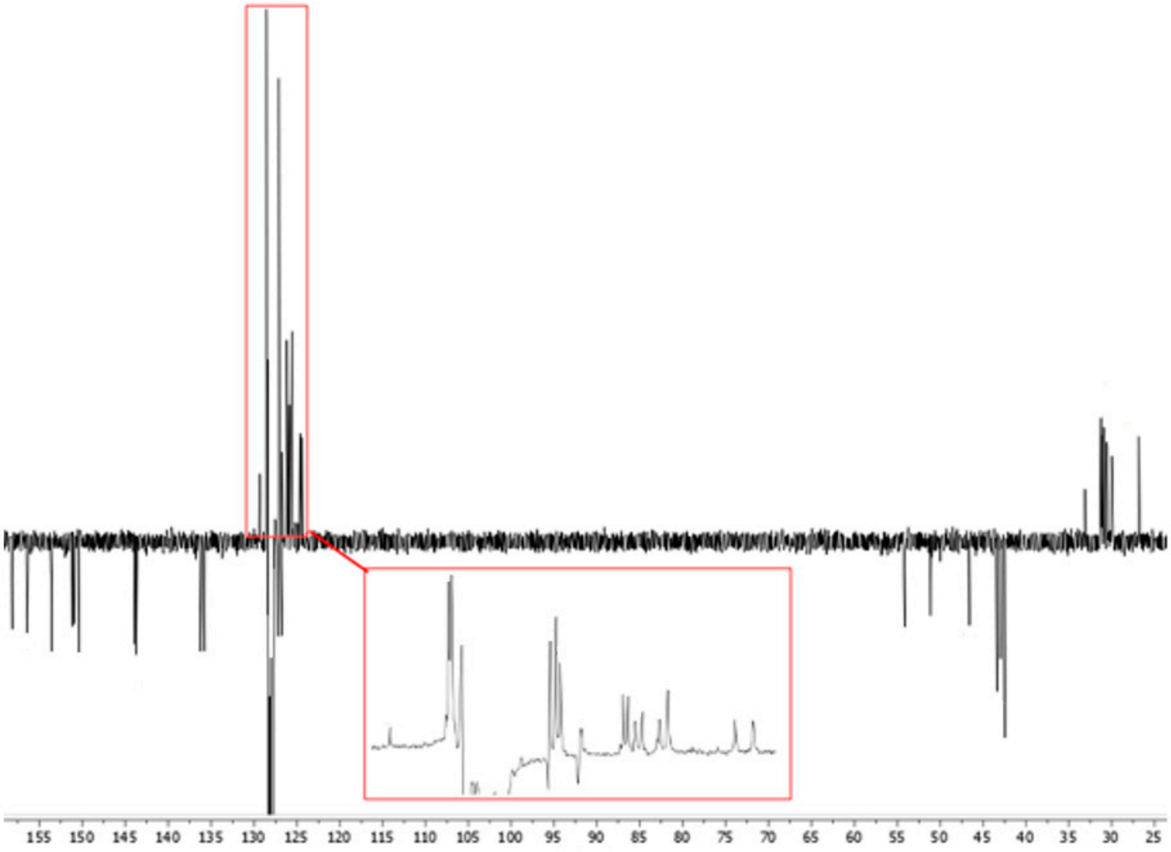

Figure S5C. ^{13}C{^{1}H} NMR spectrum of **4** in C_6D_6.

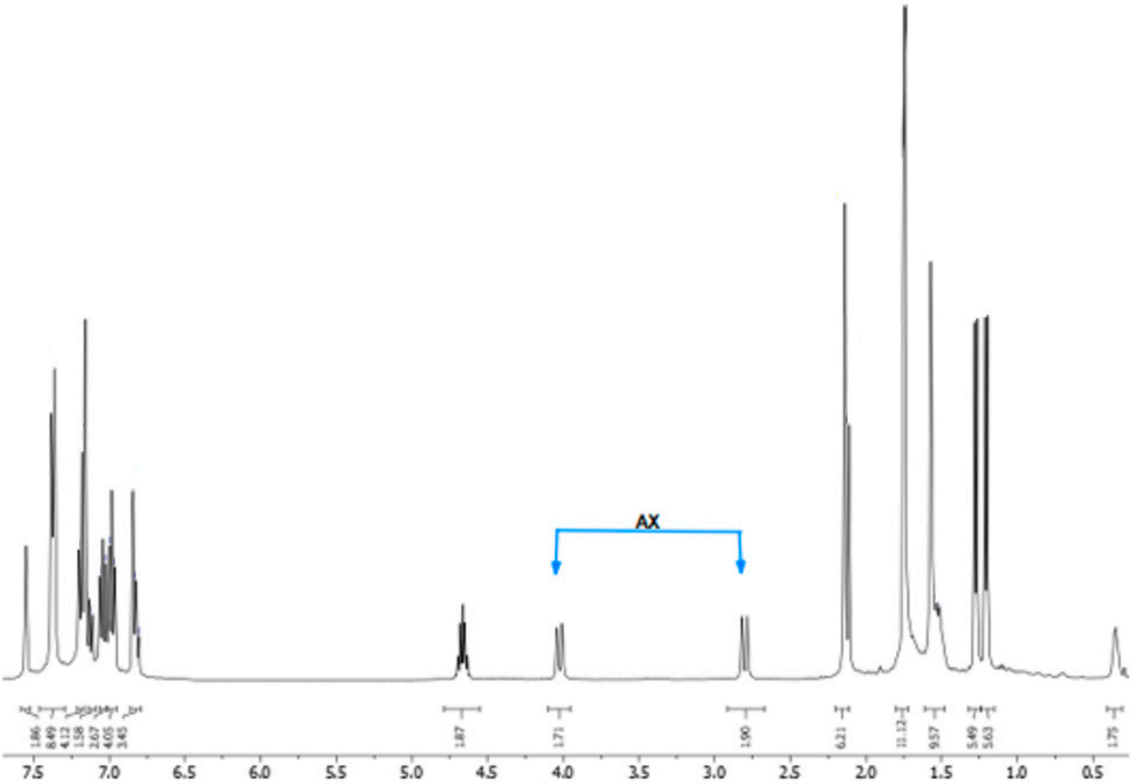

Figure S6A. ^{1}H NMR spectrum of **5** in C_6D_6.

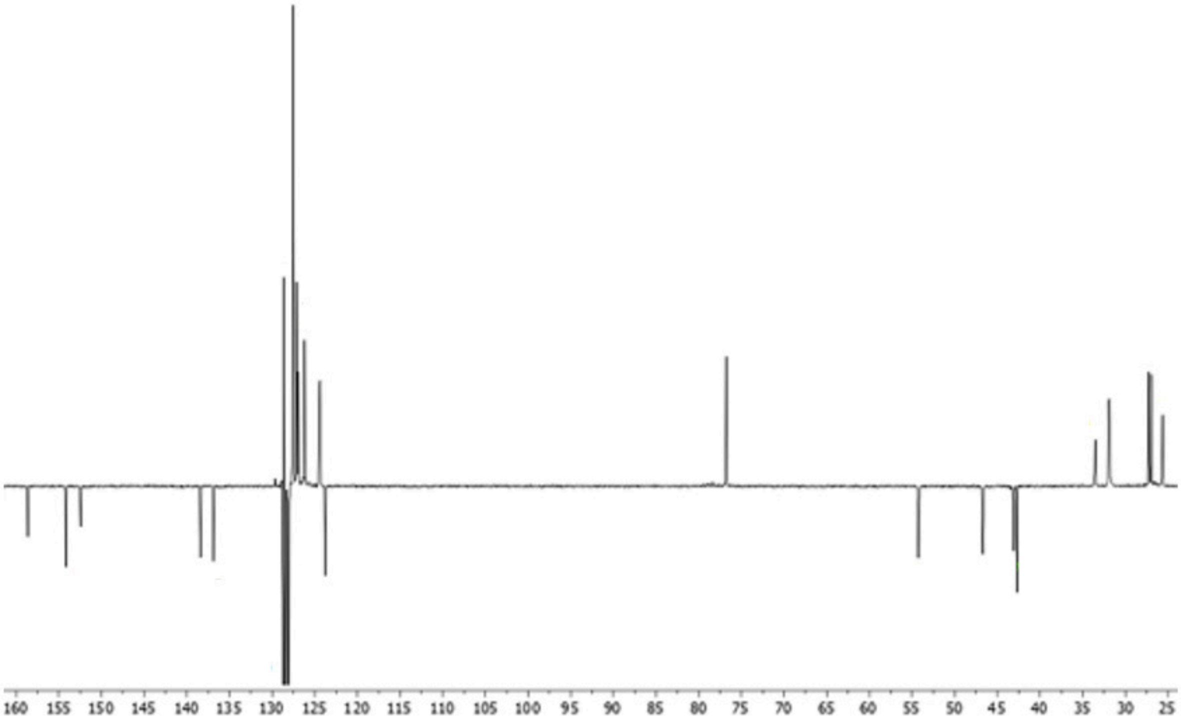

Figure S6B. $^{13}C\{^{1}H\}$ NMR spectrum of **5** in C_6D_6.

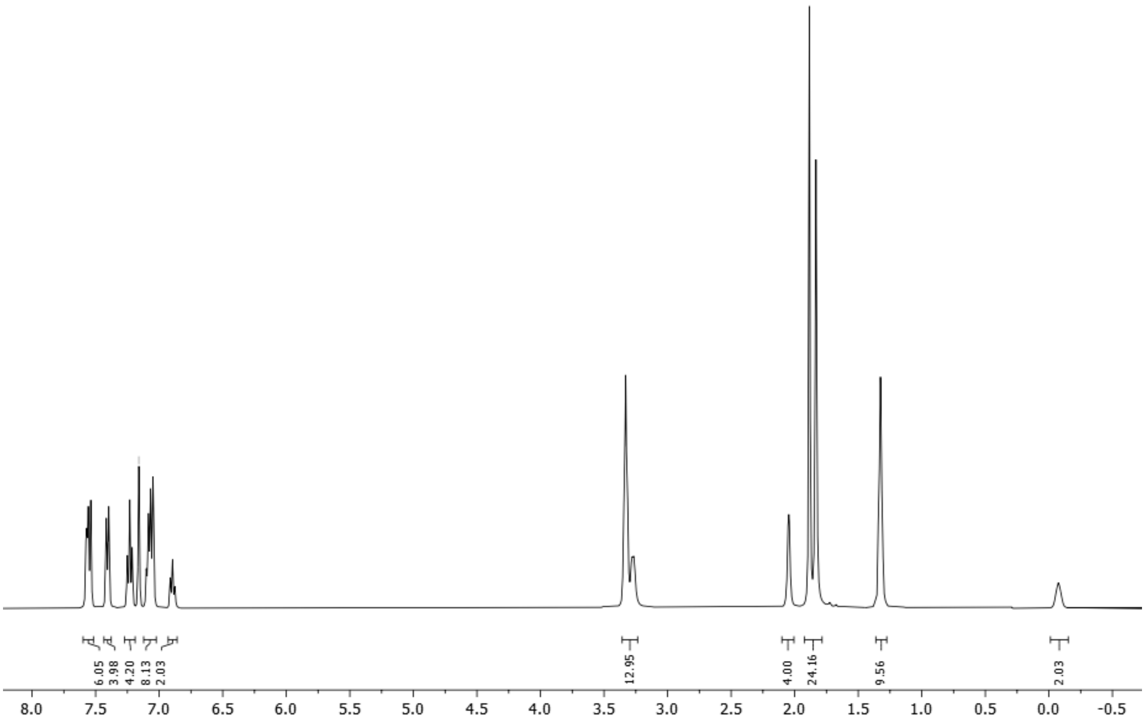

Figure S7A. ^{1}H NMR spectrum of **6** in C_6D_6.

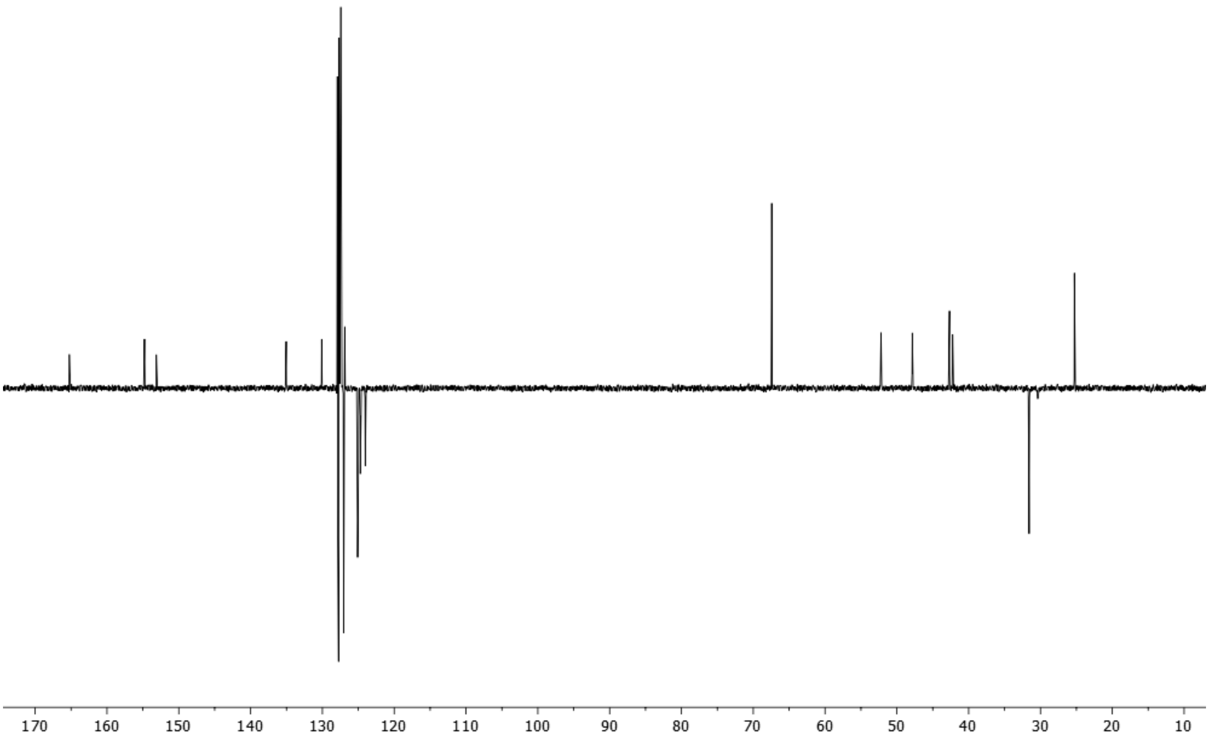

Figure S7B. $^{13}C\{^{1}H\}$ NMR spectrum of **6** in C_6D_6.

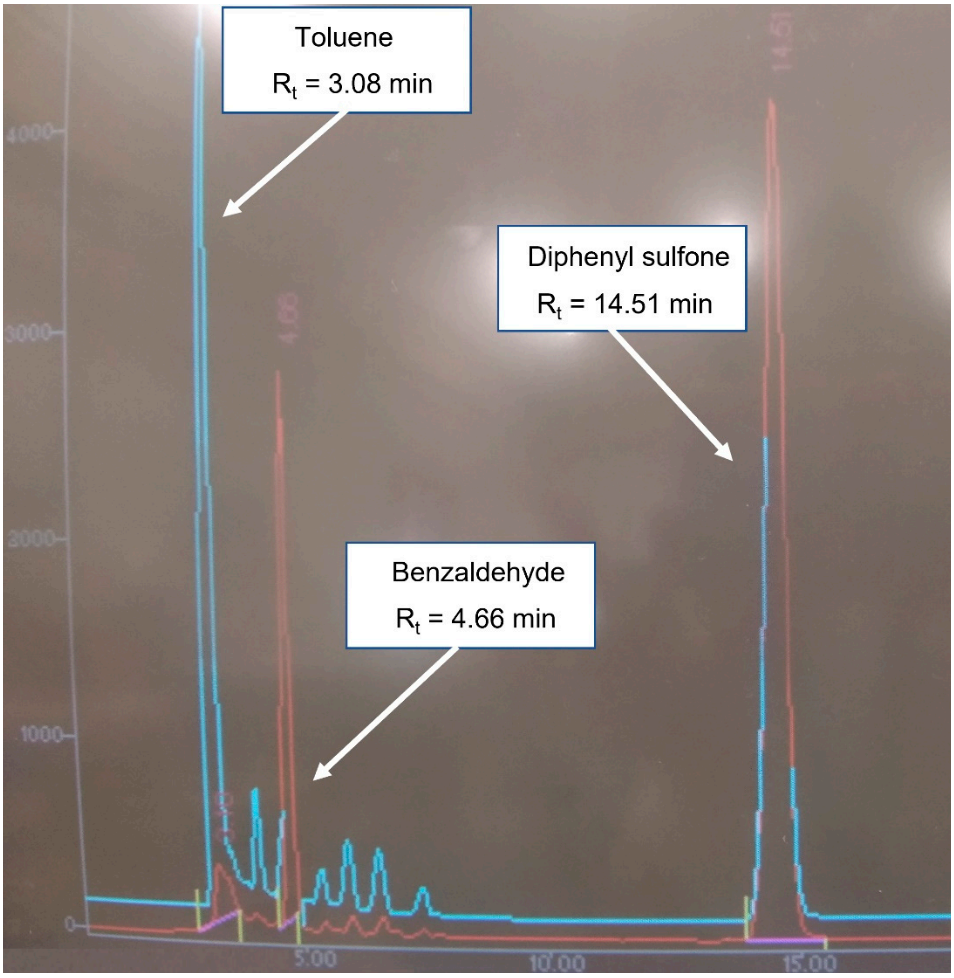

Figure S8. HPLC spectra of the products obtained after 4 h at 55 °C in the absence (blue) and in the presence (red) of TEMPO (Table 1, entries 2 and 3, respectively).
